# BRPF1 in cancer epigenetics: a key regulator of histone acetylation and a promising therapeutic target

**DOI:** 10.1038/s41420-025-02730-3

**Published:** 2025-10-06

**Authors:** Elena Alexandrova, Roberto Parisi, Marharyta Smal, Domenico Di Rosa, Alfonso Carleo, Elena Orlando, Carmela Veneri, Viola Melone, Annamaria Salvati, Roberta Tarallo, Giovanni Nassa, Alessandro Weisz, Francesca Rizzo

**Affiliations:** 1 Laboratory of Molecular Medicine and Genomics, Department of Medicine, Surgery and Dentistry “Scuola Medica Salernitana” University of Salerno, Baronissi, Italy; 2 Molecular Pathology and Medical Genomics Unit, AOU “S. Giovanni di Dio e Ruggi d’Aragona”, University of Salerno, Salerno, Italy; 3 Genome Research Center for Health—CRGS, Campus of Medicine of the University of Salerno, Baronissi, Italy

**Keywords:** Targeted therapies, Prognostic markers

## Abstract

Bromodomain and PHD finger-containing protein 1 (BRPF1) is an essential component of histone acetyltransferase complexes, where it acts as a scaffold to facilitate their assembly and enzymatic activity, thereby playing a key role in chromatin remodeling and transcriptional regulation. Emerging evidence indicates that BRPF1 is frequently dysregulated in cancer and contributes to tumorigenesis by modulating key oncogenic pathways. Its overexpression has been associated with poor prognosis in multiple malignancies, highlighting its relevance as a candidate for targeted therapy. Specifically, BRPF1 is particularly implicated in cancers of gastrointestinal and genitourinary systems, as well as in brain, skin, breast, and hematological tumors. The development of selective BRPF1 bromodomain inhibitors has opened new therapeutic avenues, with preclinical models showing notable anticancer effects. Moreover, combinatorial strategies involving BRPF1 inhibitors and other targeted therapies have shown promise in enhancing treatment efficacy. This review provides a comprehensive overview of BRPF1 structure and function, its oncogenic role, and the therapeutic targeting strategies. We also examined current advancements in drug development, highlighting the challenges in BRPF1 inhibition, and proposed future research directions to elucidate its role in cancer epigenetics and translate these insights into improved clinical outcomes.

## Facts

- BRPF1 functions as a scaffold and multivalent chromatin reader in histone acetyltransferase complexes, yet the precise mechanisms dictating its recruitment and target specificity in different physiological and pathological contexts remain incompletely understood.
- BRPF1 is frequently dysregulated in cancer, but its prognostic value is highly context-dependent, suggesting it may act as both oncogene and tumor suppressor depending on tumor type and molecular background.
- Selective BRPF1 bromodomain inhibitors show promising preclinical efficacy, but their translation into clinical use is hampered by limited pharmacokinetic and safety data, as well as incomplete knowledge of patient populations most likely to benefit.
- BRPF1 drives therapy resistance in multiple cancers through modulation of drug efflux, metabolic reprogramming, and chromatin accessibility, making it a candidate target for combinatorial therapeutic strategies.
- The interplay between BRPF1 and key oncogenic pathways (e.g., p53, ERα, Wnt, SREBP2) warrants further investigation to exploit its role in precision oncology.

## Introduction

In eucaryotic cells, the degree of chromatin compaction and therefore DNA accessibility are fine-tuned by reversible post-translational modifications (PTMs) of histone tail residues, usually referred to as histone PTMs (HPTMs). These modifications affect internucleosomal interactions, leading to chromatin remodeling by recruiting proteins capable of nucleosome repositioning and regulating essential cellular processes such as transcription, recombination, DNA repair, replication as well as genome topology [1]. Acetylation, methylation, phosphorylation, ubiquitination, and sumoylation represent well-characterized HPTMs [2], whereas other modifications have only recently been identified [3]. Aberrant patterns of HPTMs, often resulting from dysfunction of related enzyme, have been implicated in the pathogenesis of various disorders, including cancer, infectious, psychiatric, neurodegenerative, and autoimmune/inflammatory diseases [4–6]. The reversible nature of histone modifications represents a valuable target for developing therapeutic strategies aimed at early prevention and treatment of a wide range of diseases.

Bromodomain-containing proteins (BCPs) are a family of chromatin readers that recognize acetylated lysine residues on histone tails [7]. BCPs are further categorized into the bromodomain and extra-terminal (BET) and the non-BET proteins [8, 9]. While other reader families are also well-studied, bromodomains (BrDs) are especially notable due to their direct translational relevance and the possibility to modulate their activity by selective small-molecule inhibitors targeting BET BrDs, many of which have shown robust anticancer activity in preclinical studies. However, the emergence of resistance and adverse events has limited the application of BET inhibitors [10]. Moreover, growing evidence support the involvment of non-BET BCPs in inflammation, cancer and cardiovascular diseases [11]. Unlike BET proteins, non-BET members exhibit structural diversity and possess multiple functional domains, resulting in more complex biological functions. Consequently, there is increased interest in exploring non-BET BrD-containing proteins as therapeutic targets.

Among non-BET BrD-containing proteins, the bromodomain and plant homeodomain (PHD) finger-containing (BRPF) family exhibits several unique features. In particular, BRPF1/2/3 are multivalent proteins that act as the scaffolds in histone acetyltransferase (HAT) complexes [12–14], mainly responsible for histone acetylation, a widespread epigenetic modification in cells [15]. Due to its negative charge, histone acetylation neutralizes the positive charge of histone tails, reducing their affinity for the negatively charged DNA. This process promotes chromatin opening, thereby exposing regulatory DNA regions required for crucial processes such as replication and transcription [16, 17].

Among the BRPF family, BRPF1 stands out due to its broader impact on chromatin regulation and its central role in proper development and differentiation, particularly in hematopoiesis and neurogenesis. BRPF1 is required for mouse embryonic survival [18], and it regulates the development of several adult tissues, including the brain, axial skeleton, and the hematopoietic system [19–22]. It also plays a role in maintaining embryonic stem cells pluripotency [23]. *BRPF1* mutations are associated with intellectual disabilities and congenital abnormalities. In particular, BRPF1 haploinsufficiency causes Intellectual Developmental Disorder with Dysmorphic Facies and Ptosis (IDDDFP) [24, 25]. Dysregulation of BRPF1 is also associated with multiple cancer types, as detailed below.

This review discusses the role of BRPF in HAT complexes assembly and chromatin binding, recapitulates its functions in physiological conditions, and focuses on its contribution to tumorigenesis. We also evaluated the therapeutic potential of targeting BRPF1, highlighting current pharmacological advancement in specific cancer subtypes, including gastrointestinal, genitourinary, brain, skin, hematologic cancers and others. We finally discuss key challenges and future research directions in cancer epigenetics that may support the development of new therapeutic target strategies and improve clinical outcomes.

## Structure and functions of BRPF1

### Role of BRPF1 functional domains in histone acetyltransferase complexes assembly and nucleosome binding

**Fig. 1:**
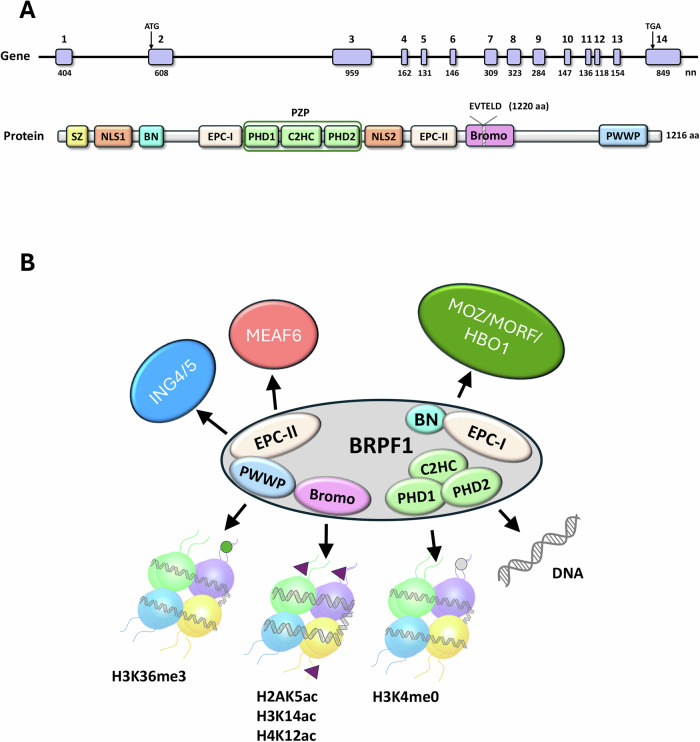
Schematic representation of BRPF1 gene structure and protein domains, and associated Histone Acetyltransferase (HAT) complexes. **A** The top panel displays the exon-intron organization of the BRPF1 gene, with ATG and TGA marking start and stop codons. Exons are represented as vertical bars, annotated with exon number and length (in base pairs, bp). The bottom panel illustrated the BRPF1 protein structure, highlighting the key domains: SZ (Sfp1-like C2H2 zinc finger), NLS1/NLS2 (nuclear localization signals), BN (BRPF1-specific N-terminal domain), EPC-I and EPC-II (enhancer of polycomb-like domains), double PHD (plant homeodomain) linked with C2HC zinc knuckle, together forming the PHD–Zinc knuckle–PHD (PZP) domain, Bromo (bromo domain) and a PWWP (chromo/Tudor-related Pro-Trp-Trp-Pro-containing) domain. **B** In the panel, the central BRPF1 protein is shown as a hub integrating various components and forming HAT complexes. BRPF1 binding partners MOZ/MORF and HBO1 interact with EPC-I domain, while ING5 and MEAF6 associate with EPC-II domain. The HAT complexes target chromatin to regulate transcription. BRPF1 recognizes various histone marks, including: H3K36me3, H2AK5ac, H3K14ac, H4K12ac, H3K4me0 and, finally, DNA.

The *BRPF1* gene, formerly known as BR140 (bromodomain protein of 140 kDa) or peregrin, was first cloned in 1994 and identified as a transcriptional regulator due to the presence of zinc finger and bromodomain motifs. In humans, BRPF1 is encoded by a 14-exone gene (Fig. 1) on chromosome 3p25, spanning ~16 kilobases and producing a protein with predicted molecular weight of 140 kDa [26]. According to UniProt database, 4 *BRPF1* isoforms are expressed, encoding proteins of 1119, 1213, 1214 and 1220 amino acids. Isoforms 1214 and 1220 correspond to BRPF1B and BRPF1A, respectively; BRPF1B encodes the functional BRPF1 protein, whereas BRPF1A contains six-residue insertion in the bromodomain that alters its structure and affects its binding capabilities [27] (Fig. 1).

BRPF1 has a dual role: it serves as a scaffold subunit essential for HAT complex assembly [12–14], and functions as a multivalent chromatin reader via several nucleosome-binding modules (Fig. 1). From N- to C-terminus, BRPF1 contains the following domains: Sfp1-like C2H2 zinc finger (SZ), nuclear localization signal (NLS) 1, BRPF1 specific N-terminal (BN) domain, enhancer of polycomb (EPC) like motif 1 (EPC-I), double PHD linked with C2HC zinc knuckle and altogether composing PHD–Zinc knuckle–PHD (PZP) domain, NLS2, EPC like motif 2 (EPC-II), a BrD and a chromo/Tudor-related Pro-Trp-Trp-Pro (PWWP) containing domain [12–14, 28, 29].

The BN domain, along with EPC-I and EPC-II motifs [12, 30], facilitates BRPF1’s bridging function in assembling the 4-subunit HAT complexes [13]. EPC-I, with contribution of BN, associates with the MYST domain of histone acetyltransferases MOZ (monocytic leukemia zinc finger protein; a.k.a. MYST3 and KAT6A), its paralog MORF (MOZ-related factor, a.k.a. MYST4 and KAT6B) and HBO1 (histone acetyltransferase binding to ORC1, a.k.a. MYST2 and KAT7), whereas EPC-II is necessary and sufficient for stable interaction with ING4/5 (inhibitor of growth 4 or 5) and MEAF6 (mammalian Esa1-associated factor-6) [12–14]. Although BRPF1 can bind all three MYST HATs, it preferentially associates with MOZ and MORF [31, 32]. Notably, the specificity of these complexes for histone H3 is mediated by BRPF1 N terminal domain homologous to the EPC proteins’EPcA region [33, 34] underscoring the role of the scaffold, rather than the acetyltransferase, in histone tail selection [14].

The PZP, Bromo, and PWWP domains perform epigenetic reader functions, enabling BRPF1 to interact with chromatin. The PZP domain is highly conserved in BRPF2/3 and JADE proteins, which are typically associated with HBO1 [31, 32, 34, 35]. Structural study demonstrated that both PHD domains are required for the MOZ-BRPF1-ING5-MEAF6 complex to bind histone H3 in vivo. The PHD1 domain harbors negatively charged residues required for binding H3 [36], while PHD2 and C2HC zinc knuckle domains mediate DNA interaction through positively charged residues (in particular, K383, K390, and R392). The PZP domain shows a preference for linker or extranucleosomal DNA, suggesting its role in the stabilization of the MORF complexes at euchromatin regions [36]. BRPF1 tightly binds to nucleosome by anchoring the histone H3 N-terminus via PHD1 and linker DNA via PHD2 domain [14, 29, 36]. BRPF1 PWWP domain instead recognizes the H3K36me3 histone mark, typically found in actively transcribed chromatin regions, as demonstrated by crystallization of the BRPF1-H3K36me3 complex [37, 38]. Finally, its BrD interacts with multiple acetylated lysines at histone N-terminal tails, particularly H2AK5ac, H4K12ac, and H3K14ac [39]. However, not all BRPF1 isoforms retain this capability: the 6-residue insert in BrD ZA-loop present in BRPF1A isoform structurally impairs its ability to bind acetylated lysines and inhibitors [27, 40–42].

### BRPF1 is a key component of histone acetyltransferase complexes and affects chromatin regulation

BRPF1 plays a pivotal role in multi-subunit HAT complexes assembly, regulating their enzymatic activity and substrate specificity. As previously mentioned, BRPF1 acts as a scaffold within MOZ/MORF and HBO1 HAT complexes, enabling the formation of a tetrameric complex that includes MOZ/MORF or HBO1, ING4/5 and MEAF6, proteins that do not directly interact without BRPF1 support [13, 14]. BRPF1 also stimulates the acetyltransferase activity of MOZ/MORF and HBO1 and limits the HBO1’s substrate specificity to histone H3 [13, 43, 44]. Additionally, BRPF1 functions as a multivalent chromatin reader directing the complexes to histone H3 modifications.

The BRPF1-containing HAT complexes comprise a writer module (the MYST domain of MOZ/MORF or HBO1) and five reader modules capable of recognizing unmodified H3, H3K14ac, H3/H4ac, H3K4me3, and H3K36me3. Two recruitment mechanisms of BRPF1-containing HAT complexes have been proposed. According to the first one, a combinatorial “histone code” consisting of aforementioned modifications is recognized simultaneously by MOZ/MORF complexes [45]. In the other, recruitment occurs sequentially, starting with the recognition of H3K4me3 or H3K36me3 at the active gene promoters by ING4/5 or BRPF1, respectively. This is followed by BRPF1 PZP domain binding to adjacent unmodified H3 tail and DNA, which then promotes MOZ/MORF HAT recruitment [14, 37, 45, 46]. These interactions result in increased H3K23ac and H3K14ac levels, promote chromatin relaxation, and enhance the transcription factors accessibility to regulatory DNA elements [14, 47]. MOZ’s C-terminal region can further stabilize transcription by interacting with multiple transcription factors [48–51]. Thus, the localization of the BRPF1-containing HAT complexes is influenced by other histone-modifying complexes [46].

The complexity of BRPF1-dependent HAT recruitment and function has made it challenging to fully elucidate their biological roles. Further studies are required to clarify how these complexes operate in the physiological and pathological context.

### BRPF1 in regulation of gene expression and key cellular processes

The functional role of BRPF1 was first described in 2008 by Laue et al. who showed that it maintains transcriptional memory through mitosis by remaining chromatin-bound during metaphase [52]. Further studies revealed that BRPF1 is essential for neurodevelopment and stem cell maintenance by modulating chromatin dynamics and regulating key developmental genes. Thus, *Brpf1* knockout in zebrafish impair cranial *Hox* gene expression [53] and disrupts pharyngeal segmentation [52]. In mice, *BRPF1* knockout lead to embryonic lethality around E9.5 [18], attributed to placenta vascular defects that compromise nutrient and gas exchange [46]. *BRPF1* deficient embryos also exhibit severe neurodevelopmental defects, including failed neural tube closure due to impaired neuron formation and migration [46]. Forebrain-specific *BRPF1* depletion induced aberrant neuronal migration and cell cycle deregulation during brain and hippocampus development [19, 20]. These developmental issues are thought to arise from defects in proliferation and cell cycle progression, and not from apoptosis or DNA damage events [46]. In forebrain excitatory neurons, *Brpf1* knockout reduced the frequency of miniature excitatory postsynaptic currents and downregulated genes involved in neural development, synapse function, and memory (e.g., *Pcdhgb1*, *Slc16a7*, *Robo3*, and *Rho)* [54]. In human, germline *BRPF1* mutations cause less severe but consistent phenotypes, such as congenital anomalies and IDDDFP, characterized by psychomotor and language delay, intellectual disability, and facial dysmorphisms [55–59].

The role of BRPF1 in stem cell maintenance was first described by You et al. who demonstrated that its depletion impairs hematopoietic stem cell self-renewal in mice [21]. BRPF1 knockout led to early lethality caused by bone marrow failure and aplastic anemia, associated with reduced expression of multipotency genes (e.g. *Slamf1*, *Mecom*, *Hoxa9*, *Hlf*, *Gfi1*, *Egr*, *Gata3*) and dramatic reduction of H3K23 acetylation [21]. Consistently, a patient with IDDDFP and a *BRPF1* mutation showed hematological abnormalities, such as anemia and thrombocytopenia, suggesting BRPF1’s relevance also in human hematopoiesis [60].

Finally, in human embryonic stem cells (hESCs), *BRPF1* knockout induces morphological changes typical of differentiation and impairs self-renewal. *BRPF1*-null stem cells are characterized by up-regulation of developmental genes (*SOX1*, *RUNX1* and *SOX17)* and down-regulation of pluripotent factors (*OCT4*, *NANOG,* and *SOX2)*. Notably, BRPF1 loss causes an immediate depletion of H3K23ac and a progressive loss of pluripotency [23].

Taken together, the structural organization and domain-specific functions of BRPF1 support its role as a critical scaffold and chromatin reader within histone acetyltransferase complexes such as those formed with MOZ, MORF, or HBO1. By coordinating histone acetylation and nucleosome targeting, BRPF1 fine-tunes chromatin accessibility and gene expression. Its essential contribution to embryonic development, neurogenesis, and stem cell identity highlights its function as a central regulator of epigenetic programming in both physiological and developmental contexts.

## BRPF1 in tumorigenesis

### BRPF1 expression and prognostic significance in cancer

**Fig. 2:**
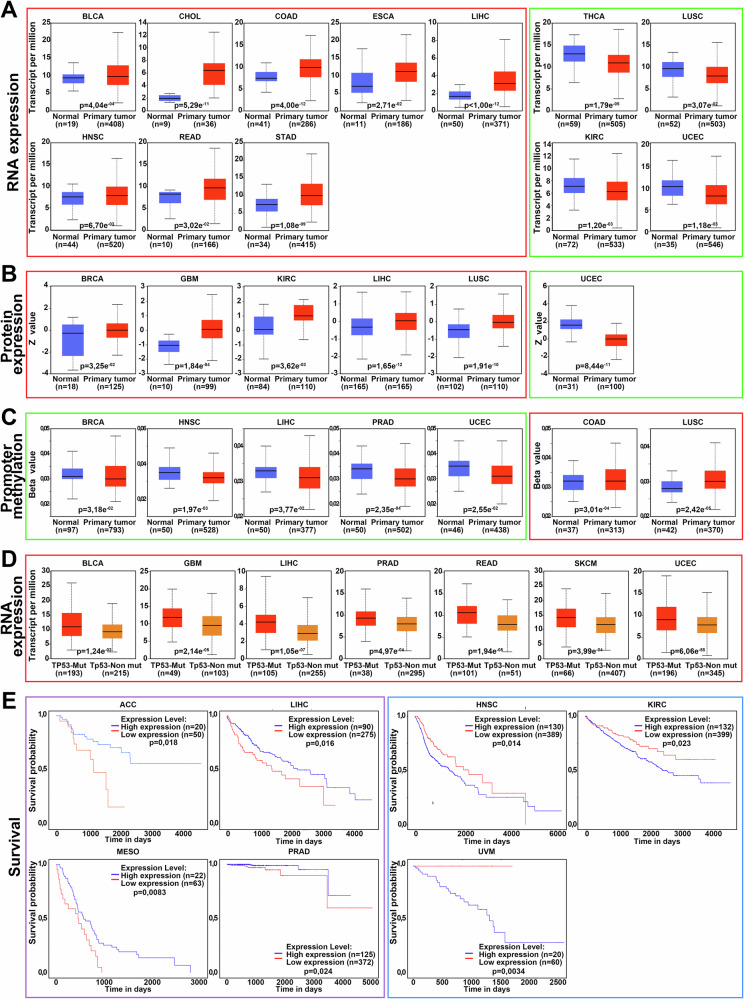
BRPF1 expression patterns and clinical relevance across various cancer types. The figure summarizes data on BRPF1 mRNA and protein expression, promoter methylation, and patient survival analysis available in The Cancer Genome Atlas (TCGA) and the Clinical Proteomic Tumor Analysis Consortium (CPTAC) databases. Each section presents specific types of analysis. Box plots in (**A**, **B**) represent BRPF1 mRNA expression (in transcripts per million) and protein expression (in Z-scores), respectively, in normal *versus* tumor tissues. Box plots indicating upregulation or downregulation of BRPF1 mRNA or protein in tumors compared to normal tissues are outlined in red and green frames, respectively. **C** shows box plots of BRPF1 promoter methylation levels (beta values) in normal and tumor tissues. Hypomethylation and hypermethylation of the BRPF1 promoter in tumors relative to normal tissues are highlighted in green and red frames, respectively. **D** displays box plots of BRPF1 mRNA expression (in transcripts per million) in *TP53*-mutated and wild type tumors. **E** shows Kaplan–Meier survival curves illustrating the correlation between BRPF1 expression levels and overall patient survival. Associations between high BRPF1 expression and poor prognosis are outlined in violet frames, while correlations with favorable survival outcomes are highlighted in blue frames. For each comparison, the number of samples in each group (n) and *p* values indicating statistical significance are provided. Only comparisons with a *p* value less than 0.05 were considered statistically significant and included in the figure. ACC Adreno Cortical Carcinoma, BLCA BLadder urothelial CArcinoma, BRCA BReast invasive CArcinoma, CHOL CHOLangiocarcinoma, COAD COlon ADenocarcinoma, ESCA ESophageal CArcinoma, GBM GlioBlastoma Multiforme, HNSC Head and Neck Squamous cell Carcinoma, KIRP KIdney Renal Papillary cell carcinoma, LIHC LIver Hepatocellular Carcinoma, LUSC LUng Squamous cell Carcinoma, MESO MESOthelioma, PRAD PancReatic ADenocarcinoma, READ REctum ADenocacinoma, SKCM SKin Cutaneous Melanoma, STAD STomach ADenocarcinoma, THCA THyroid CArcinoma, UCEC Uterine Corpus Endometrial Carcinoma, UVM UVeal Melanoma.

Recently, Cevatemre et al. reported that BRPF1 expression is significantly elevated in tumors characterized by local recurrence and metastasis, and that high BRPF1 levels correlate with reduced patient overall survival [61]. To further assess the therapeutic and prognostic potential of BRPF1 in human cancers, we queried The Cancer Genome Atlas (TCGA, https://www.cancer.gov/tcga) and the Clinical Proteomic Tumor Analysis Consortium (CPTAC) databases using the University of ALabama at Birmingham CANcer (UALCAN) data analysis portal [62, 63]. Data on BRPF1 mRNA and protein expression, as well as prognostic value in different tumor types are summarized in Fig. 2.

Comparative analysis of *BRPF1* mRNA levels in primary tumors versus normal tissues revealed that cholangiocarcinoma (CHOL), LIHC and esophageal carcinoma (ESCA) display significantly elevated *BRPF1* expression. Moderate elevation was observed in colon and stomach adenocarcinoma, while only a subset of tumors showed upregulation in head and neck squamous cell carcinoma, bladder urothelial carcinoma and rectum adenocarcinoma (Fig. 2A). At protein level, BRPF1 overexpression was evident in breast cancer (BC), glioblastoma multiforme (GBM), LIHC, and lung squamous cell carcinoma (LUSH) (Fig. 2B). These findings are consistent with prior reports, including BRPF1 overexpression in LIHC [64], low-grade gliomas [65], luminal like BC and melanoma [66–68].

Given that gene expression can be regulated by promoter methylation, where hypermethylation typically silences transcription and hypomethylation enhances gene expression, we evaluated the BRPF1 promoter methylation status. Our analysis revealed that promoter hypomethylation could drive BRPF1 overexpression in HNSC and LIHC (Fig. 2C). Interestingly, although BRPF1 transcriptional changes were not significant in breast and prostate cancer, promoter hypomethylation was still observed. Lastly, in colon adenocarcinoma some tumors exhibit BRPF1 promoter hypermethylation.

According to the present literature data, the overexpression of BRPF1 in cancer cells may be driven by tumor-specific factors such as transcription factor SP1, the Wnt/β-catenin coactivator Pygo2, and hormonal signals including prolactin [64, 69, 70]. Moreover, BRPF1 upregulation may be induced by loss or mutation of tumor suppressor genes *PTEN* and *TP53* [64, 69, 71] or protein stabilization through deubiquitination by USP35 [72]. Finally, environmental factors such as exposure to endosulfan, a genotoxic pesticide, may also increase BRPF1 expression [73].

Considering the high prevalence of *TP53* mutations in cancer and emerging evidence that mutant p53 can regulate BRPF1 [74, 75], we compared BRPF1 mRNA expression in *TP53* wild-type and mutant tumors using TCGA data. Several solid tumors, including bladder urothelial carcinoma, glioblastoma multiforme, liver hepatocellular carcinoma, prostate adenocarcinoma, rectum adenocarcinoma, skin cutaneous melanoma and uterine corpus endometrial carcinoma displayed elevated BRPF1 expression in the presence of *TP53* mutations (Fig. 2D), in agreement with the previously published data [64]. This supports a potential regulatory link between mutant p53 and BRPF1, warranting further investigation into how p53 dysfunction may influence BRPF1-mediated epigenetic changes in tumorigenesis.

Survival analysis of cancer patients data from TCGA revealed that high *BRPF1* expression represents an unfavorable prognostic factor in adrenocortical carcinoma, liver hepatocellular carcinoma, mesothelioma and prostate adenocarcinoma (Fig. 2E). These data are in line with prior studies showing reduced overall and disease-free survival of LIHC patients [64] and a shorter disease-free survival of prostate adenocarcinoma patients [72]. Moreover, high BRPF1 expression was reported as negative prognostic factor for luminal-like BC, ovarian cancer (OC) and metastatic melanoma patients [68, 76, 77].

Despite substantial evidence linking BRPF1 to cancer progression, some studies indicate potential antitumor functions, suggesting that BRPF1 may play a dual role in tumorigenesis. Thus, according to TCGA and CPTAC databases, *BRPF1* mRNA expression is slightly downregulated in thyroid carcinoma, lung squamous cell carcinoma, kidney renal clear cell carcinoma and uterine corpus endometrial carcinoma (Fig. 2A), whereas BRPF1 protein expression is slightly downregulated in clear cell renal carcinoma and more significantly in uterine corpus endometrial carcinoma compared to the respective normal tissues (Fig. 2B). To date, no mechanisms of BRPF1 downregulation have been described, except for two studies showing that cytokine TNFα and the bradykinin antagonist BKM-570 inhibit BRPF1 activity [78, 79]. In lung squamous carcinoma, BRPF1 promoter hypermethylation likely accounts for its decreased expression (Fig. 2C). In contrast, in uterine corpus endometrial carcinoma (UCEC), BRPF1 expression is reduced at both mRNA and protein levels despite promoter hypomethylation, suggesting that further research is needed to fully elucidate possible alternative regulatory mechanisms (Fig. 2C).

Interestingly, high *BRPF1* levels correlate with better prognosis in some cancers, including head and neck squamous carcinoma, kidney renal clear cell carcinoma and uveal melanoma (Fig. 2D), suggesting a context-dependent role for BRPF1 in cancer biology.

In summary, BRPF1 expression varies widely across cancer types and contexts, and further research is needed to fully define its prognostic and predictive value.

### BRPF1 in regulation of key processes in cancer cells

BRPF1 has been implicated in regulating multiple processes critical for cancer progression, including cell growth, colony formation, stemness, cell cycle control, apoptosis, senescence, metabolism, invasiveness, and DNA damage response. Several studies have shown that BRPF1 pharmacological inhibition suppresses proliferation in different tumors, including LIHC [64], colon adenocarcinoma (COAD) [69], high grade serous ovarian cancer (HGSOC) [76, 80], high grade glioma (HGG) [65], BRAF-mutant melanoma [77], Epstein–Barr virus (EBV)-positive nasopharyngeal carcinoma (NPC) [81] and both AntiEstrogen (AE)-responsive and -resistant BC [68], whereas *BRPF1* knockdown was shown to inhibit the growth of HGSOC and both taxane-sensitive and resistant Prostate Cancer (PCa) [61, 80]. Beyond proliferation, colony formation assay demonstrated that genetic or pharmacological BRPF1 inhibition reduces clonogenicity in LIHC, HGSOC, COAD and acute myeloid leukemia (AML) [64, 69, 76, 82], while BRPF1 overexpression enhances clonogenic growth in PCa [72]. In LIHC, BRPF1 sustains liver cancer stem cells, by promoting the expression of NOTCH1, OCT4, and EPCAM [64].

BRPF1 also regulates cell cycle progression, with pharmacological inhibition causing arrest at either G1 or G2/M phase, depending on tumor context. G1 arrest was observed in LIHC and AE-sensitive and -resistant BC cells [64, 68], while G2/M blockade occurred in taxane-resistant PCa and melanoma [61, 83]. In HGSOC, both arrest types were reported [76]. With regard to cell death, BRPF1 inhibition promoting apoptosis in HGSOC and BC [68, 76], while senescence in LIHC [64]. In OC, *BRPF1* knockdown also suppressed cell viability and anaerobic metabolism, as shown by reduced glucose consumption and lower ATP and lactate levels [80]. In addition, BRPF1 inhibition reduced invasiveness, delayed wound healing and increased DNA damage of HGSOC cells [76].

These findings suggest that BRPF1 modulates key oncogenic pathways, a topic further discussed in the following section.

### BRPF1 in regulation of oncogenic signaling pathways

BRPF1 plays a crucial role in regulating oncogenic signaling pathways by modulating chromatin accessibility and activating the transcription of cancer-related genes. It has been identified as a key regulator of oncogenes E2F2 and EZH2 [64], as well as transcription factors that drive tumorigenesis [84]. BRFP1 also influences Wnt signaling [80], estrogen signaling via ESR1 [68], and cooperates with CDC7 to regulate MYC and IGF1R pathways [83].

Its role in maintaining cellular stemness extends from normal to cancer cells, where BRPF1 contribute by modulating chromatin accessibility of stemness-associated genes [85], including *NOTCH1*, *OCT4*, *EPCAM,* and *HOX* genes [64, 82]. BRPF1 is also implicated in cancer cell metabolism by upregulating the transcription factor SREBP2, which promote the expression of genes involved in the mevalonate (MVA) metabolism [72], NAD signaling, and PPARα-driven lipid metabolism [76]. Furthermore, BRPF1 increases expression of drug efflux transporter ABCB1/P-glycoprotein, contributing to multidrug resistance [61, 72, 86]. BRPF1 has also been linked to the regulation of ribosome biogenesis and global protein synthesis [86], and emerging evidence suggests a role in modulating immune cell infiltration within the tumor microenvironment [80].

Altogether, current evidence highlights BRPF1 as a context-dependent regulator of tumorigenesis. While its overexpression is frequently associated with poor prognosis and aggressive clinical behavior, especially in liver, breast, prostate, and ovarian cancers, its expression patterns and prognostic impact vary across tumor types. Functional studies support a central role for BRPF1 in promoting proliferation, stemness, and therapy resistance by coordinating chromatin remodeling and activating oncogenic transcriptional programs. These findings consolidate BRPF1 as a clinically relevant biomarker and therapeutic target, highlighting the need for further investigations of its role in specific cancer settings.

## BRPF1 as a therapeutic target

### Development of specific BRPF1 bromodomain inhibitors

The bromodomain acetyl-lysine binding pocket represents an excellent target for developing small-molecule inhibitor that disrupt protein interactions [87]. While most drug discovery efforts have focused on targeting BET bromodomains, leading to numerous inhibitors, some of which are currently in clinical trials (summarized by Guo et al. and Kumar Gajjela et al. [10, 88]), increasing interest has shifted toward non-BET bromodomains, including BRPF1, whose druggability has now been established [88].

**Fig. 3:**
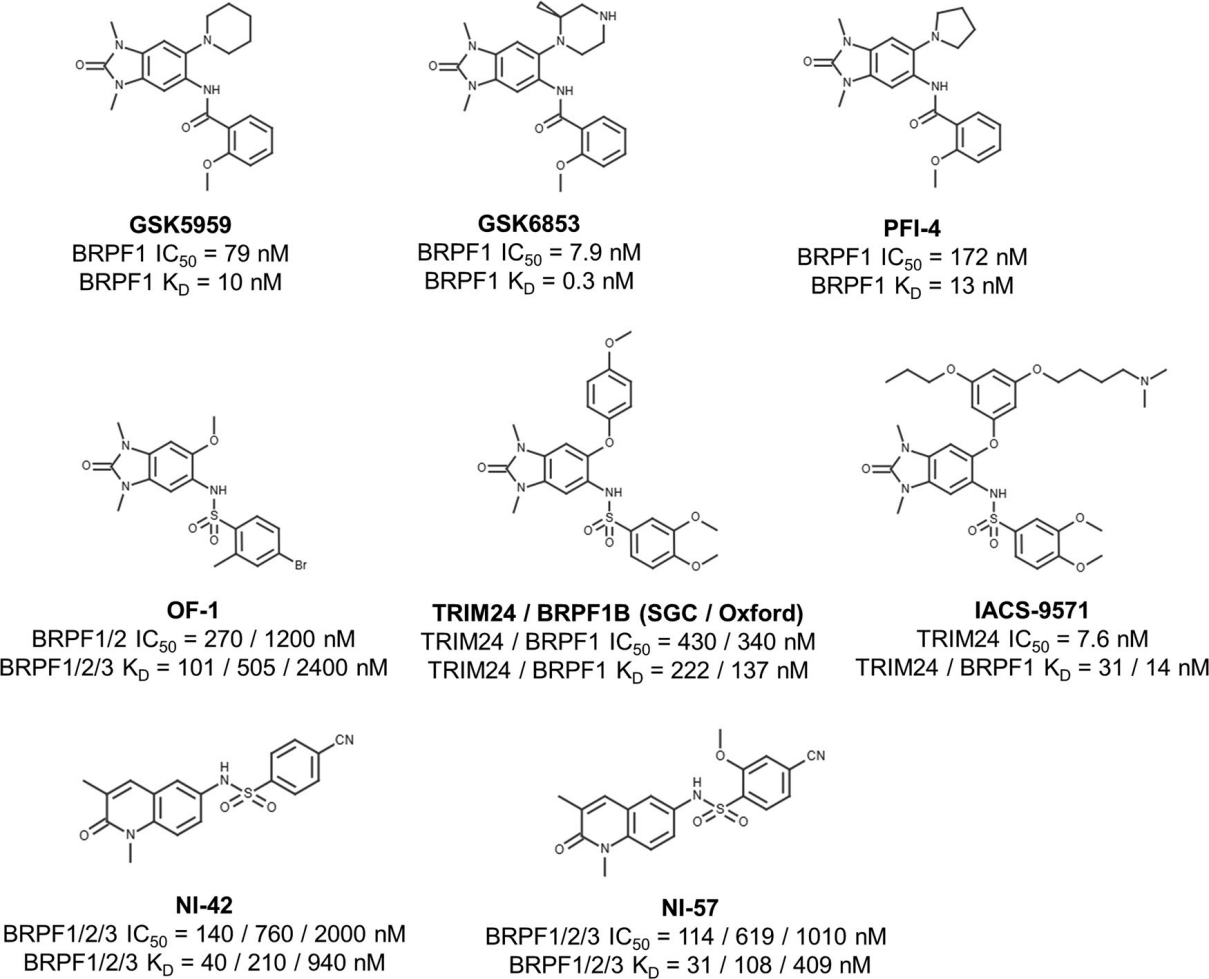
Structures and pharmacological propriety of currently available small-molecule BRPF1inhibitors. Selective inhibitors (GSK5959, GSK6853, PFI-4), pan-BRPF inhibitors (OF-1, NI-42, NI-57), and dual TRIM24/BRPF1 inhibitors (SGC/University of Oxford, IACS-9571) are shown. Potency (IC_50_) and binding affinity (K_D_) are indicated below the chemical structure of each compound.

Given BRPF1’s essential role in chromatin remodeling and transcriptional regulation, it has become a promising target in cancer therapy. Several small-molecule inhibitors that antagonize BRPF1 functions have been developed (Fig. 3). These compounds, bearing either 1,3-dimethylbenzimidazolone or N-methylquinolin-2-one scaffolds, prevent BRPF1’s interaction with acetylated histones, reducing its chromatin-binding affinity [41, 89]. These probes function as selective BRPF1 bromodomain inhibitors or pan-BRPF inhibitors, though most exhibit preferential activity toward BRPF1.

The first BRPF1B-selective inhibitor, GSK5959, exhibited strong binding affinity (pIC50 = 7.1, pKD = 8.0), with 90-fold selectivity over BRPF2 and >500-fold over BET family members [40]. Selectivity was attributed to structural differences, such as the presence of Pro658 in BRPF1 instead of Ser592 in BRPF2 or Asn619 in BRPF3.

Further optimization led to the synthesis of GSK6853, a benzimidazolone analogue with enhanced potency (pIC50 = 8.1, pKD = 9.5), improved aqueous solubility (140 mg/mL), over 1600-fold selectivity against all other bromodomains tested, and excellent in vivo suitability, (85% bioavailability in mice) [41].

The Structural Genomics Consortium (SGC), in collaboration with Pfizer and University College London, reported four BRPF inhibitors: one highly selective BRPF1B inhibitor (PFI-4) and three pan-BRPF inhibitors (OF-1, NI-42, and NI-57), which exhibited limited selectivity among BRPF family members. Dose-response ALPHAscreen assays showed IC50 values of 172 nM, 270 nM, 140 nM, and 114 nM for PFI-4, OF-1, NI-42, and NI-57, respectively [27, 42, 90].

PFI-4, a close analogue of GSK5959, selectively bound BRPF1B (KD = 13 nM), but not the BRPF1A isoform. Similarly, the compound OF-1 failed to inhibit BRPF1A but bound BRPF1B (KD = 100 nM), BRPF2 (KD = 500 nM), and BRPF3 (KD = 2400 nM), displaying 39-fold selectivity over BRD4 [27].

Pan-BRPF inhibitors NI-42 and NI-57 featured a structurally distinct scaffold (Fig. 3), N-methylquinolin-2-one, and exhibited excellent selectivity over non-class IV BRD proteins (>32-fold) [42, 90], and strong binding to BRPF1 (KD = 40/31 nM) and BRPF2 (KD = 210/108 nM) over BRPF3 (KD = 940/409 nM). These compounds moderately inhibited proliferation in selected cancer cell lines, such as MLL-rearranged acute leukemias, colon, lung, gastric, and hepatocellular cancers, with minimal cytotoxicity. Pharmacokinetic data from mice indicated their suitability for in vivo studies, with NI-42 exhibiting better oral bioavailability (49%) despite incomplete absorption [42, 90].

In parallel, two groups independently reported the discovery of dual TRIM24/BRPF1 inhibitor based on a benzimidazolone scaffold (Fig. 3). One such compound, developed by SGC and the University of Oxford, bound TRIM24 and BRPF1 with KD values of 222 nM and 137 nM, respectively. This compound showed low cytotoxicity across a panel of cancer cell lines, with inhibitory effect observed only in the MM.1S myeloma cell line [91].

The second dual inhibitor, IACS-9571, demonstrated even stronger binding, with a KD value of 31 nM for TRIM24 and 14 nM for BRPF1. However, its selectivity within the BRPF family was limited, with only 9- and 21-fold selectivity over BRPF2 and BRPF3, respectively. Despite this, IACS-9571was considered suitable for in vivo application due to its excellent solubility (76 mM) and oral bioavailability of 29% in mice [89, 92].

### BRPF1 inhibitors as anticancer drugs

BRPF1 inhibitors have demonstrated promising potential in preclinical cancer studies. Initial evidence came from osteoclastogenesis research, where PFI-4, OF-1, and NI-57 impaired RANKL-induced differentiation of monocytes into bone-resorbing osteoclasts without affecting the viability of control cells. These compounds also suppressed MMP9 secretion, a key enzyme involved in stromal remodeling and bone metastasis, thus suggesting a role in limiting cancer progression in bone [27]. A recent study using a structurally distinct BRPF1B inhibitor based on 3-acetylindole scaffold further confirmed its ability to downregulate osteoclast markers and block RANKL-induced bone resorption via the NFATc1–c-fos pathway [93].

Although earlier reports suggested that OF-1 had a higher affinity for BRPF1B [27], structural studies by He et al. revealed preferential binding of OF-1 to the BRPF1A bromodomain via stable hydrophobic interactions [94]. This interaction significantly promotes the expansion of Lin−Sca-1+c-Kit+ hematopoietic stem and progenitor cells (HSPCs) ex vivo without affecting their differentiation potential. Selective targeting of BRPF1A by OF-1 increased histone acetylation and chromatin accessibility, thereby activating self-renewal genes [94]. These findings highlight the therapeutic potential of BRPF1A-selective inhibitors for enhancing HSPC expansion in clinical applications, including transplantation in hematologic malignancies.

GSK5959 has shown antitumoral activity in vitro in LIHC, colon cancer, HGSOC, and estrogen responsive (ER) BC [64, 68, 69, 76]. Similarly, GSK6853, an optimized analog of GSK5959, significantly reduced tumorigenic properties in high-grade glioma, ER BC, and HGSOC [65, 68, 76]. OF-1 and NI-57 also displayed strong antitumor effect in LIHC [64]. A detailed discussion of the effects of these compounds across different cancer types will be presented in disease-specific sections of this review.

Importantly, the efficacy of GSK5959 and GSK6853 has been assessed in preclinical models. GSK5959 significantly inhibited subcutaneous LIHC tumor growth in vivo without evident toxicity and showed antitumor activity on Pygo2-high colon adenocarcinoma [64, 69], while GSK6853 exhibited marked antitumor activity in ex vivo patient-derived ER BC models, demonstrating the clinical translational potential of BRPF1-targetd therapies [68].

### Application of BRPF1 inhibitors in combinatorial cancer therapy

Impairment of cell signaling pathways by BRPF1 inhibitors may expose tumor vulnerabilities, enabling the strategic use of additional agents in combinatorial therapy. Several studies have explored the synergistic potential of combining BRPF1 inhibitors with other drugs. In one study on triple-negative BC, Wu et al. proposed that inhibiting glycolysis could induce epigenetic sensitivities to BRD inhibitors [95]. Co-treatment of triple-negative BC cells with the GLUT1 inhibitor, BAY-876, and OF-1 led to a marked reduction in cell viability, mainly through apoptosis, compared to monotherapies. Notably, the BRPF1-selective inhibitor PFI-4 and the more potent NI-57, when combined with BAY-876, failed to replicate the OF-1’s effect [95].

Another study investigated GSK6853 in ER BC and found that combining it with AEs (TAM or ICI) or the CDK4/6 inhibitor Palbociclib resulted in synergistic growth-inhibition at suboptimal doses. These effects were observed in both AE-sensitive and -resistant BC cells [68].

Recent finding also shows that PFI-4 and OF-1 sensitize Taxol-resistant triple-negative BC cells by downregulating ABCB1 (P-glycoprotein), a key efflux transporter involved in multidrug resistance [86]. The combination of Taxol with PFI-4 and OF-1 significantly impaired the viability of resistant cells (T1-160 and T2-450), while having minimal impact on their sensitive parental counterparts. In a related study using GSK5959, PFI-4, and OF-1, the same research group demonstrated that castration-resistant prostate cancer cells depend on BRPF1 for resistance to docetaxel and cabazitaxel, implicating BRPF1in the regulation of ABCB1, mTOR, and unfolded protein response (UPR) signaling [61].

Moreover, dual BET/BRPF1 inhibitors with acetylpyrrole scaffold demonstrated greater efficacy than either the standalone BET (JQ1) or BRPF1 (GSK6853) inhibitor in inducing growth arrest and cellular death in liver cancer cells Huh7 [96].

Taken together, the development of BRPF1 inhibitors has reached a level of maturity that supports their further exploration as therapeutic agents. Selective and pan-BRPF1 compounds, including dual bromodomain inhibitors, have shown favorable pharmacokinetic properties, preclinical efficacy across multiple cancer models, the capacity to enhance treatment responses and to overcome chemotherapy resistance. Their ability to modulate BRPF1-dependent transcriptional programs while sparing normal cells underscores their potential in targeted cancer therapy.

## BRPF1-mediated oncogenic mechanisms in cancer

### The role of BRPF1 in gastrointestinal cancers

**Fig. 4:**
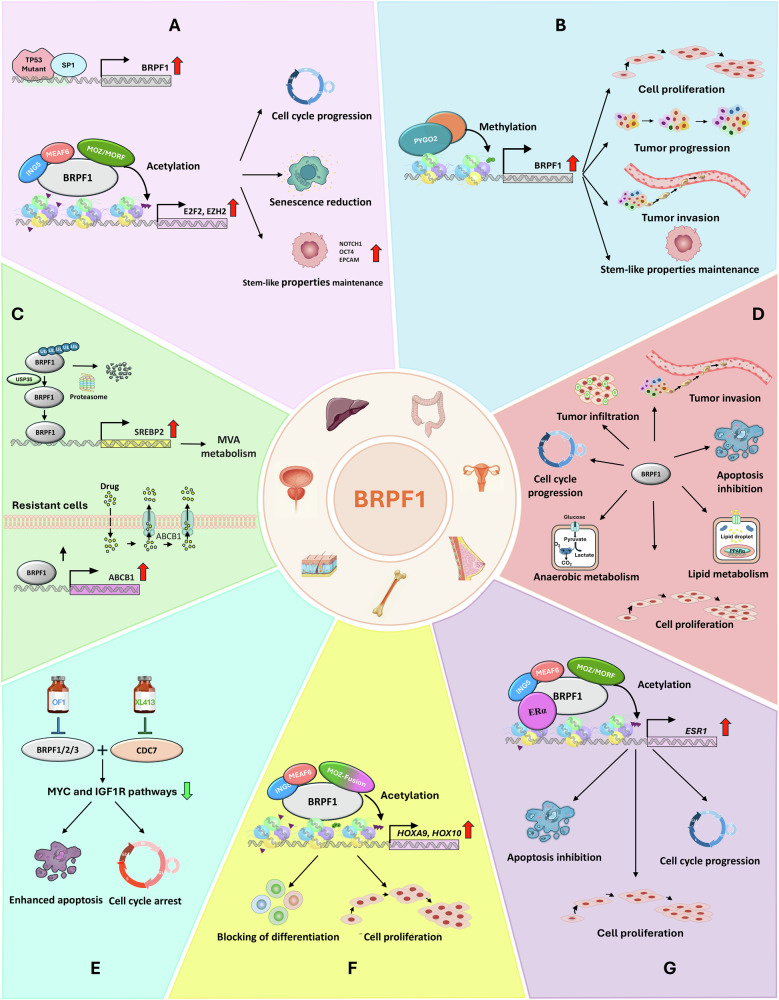
An overview of BRPF1-mediated oncogenic mechanism in various cancers. Schematic representation of BRPF1-regulated cellular processes implicated in progression of liver (**A**), colon (**B**), prostate (**C**), ovarian (**D**), skin (**E**), hematologic (**F**) and breast (**G**) cancers. **A** Shows that in hepatocellular carcinoma, aberrant activity of p53 or other transcription factors drives BRPF1 overexpression resulting in histone acetylation, chromatin remodeling, and increased expression of E2F2 and EZH2. This, in turn, promotes tumor progression by enhancing cell cycle progression, maintaining cancer cell stemness and reducing cellular senescence. **B** illustrates that Pygo2 binds to the BRPF1 promoter on chromatin, driving its overexpression and promoting tumor progression in colon adenocarcinoma cells through increased proliferation and the maintenance of stem cell-like properties. **C** outlines a mechanism whereby BRPF1 accumulation in prostate cancer (PC) cells results from its stabilization by USP35-mediated deubiquitination, leading to increased H3K4me3 levels and enhanced transcription of SREBP2. This drives mevalonate metabolism, promoting cancer cell propagation and stemness. Additionally, the panel highlights BRPF1’s role in the development of taxane resistance in PCa by regulating ABCB1-mediated drug efflux and promoting the cell cycle of taxane-resistant cells. **D** highlights the role of BRPF1 in enhancing the invasive and proliferative potential of ovarian cancer (OC) cells through increased anaerobic metabolism and activation of Wnt signaling. It also outlines how BRPF1 inhibition leads to cell cycle disruption, elevated apoptosis, and increased DNA damage. **E** highlights the anticancer effects of the combined inhibition of CDC7 and BRPF1 using XL413 and OF1 in melanoma, respectively, resulting in the suppression of MYC and IGF1R signaling pathways, increased apoptosis, and induction of cell cycle arrest. **F** illustrates the role of BRPF1 in acute myeloid leukemia (AML), where its activity leads to elevated H3 acetylation levels and transcriptional activation of the *Hoxa9* and *Hoxa10* genes, thereby promoting the stemness of AML cells. **G** depicts the oncogenic role of BRPF1 in both AE-sensitive and -resistant breast cancer (BC) cells through transcriptional regulation of the estrogen signaling pathway. It also shows that BRPF1 inhibition suppresses proliferation, disrupts the cell cycle, and promotes apoptosis in BC cells.

Various lines of evidence suggest that BRPF1 functions as an oncogene in LIHC where its expression was found to be upregulated. Cheng et al. [64] suggested that BRPF1 overexpression may be driven by the transcription factor SP1, whereas other studies demonstrated the role of mutant p53 in regulating BRPF1 in LIHC cells (Fig. 4A). Specifically, *TP53* mutations, a canonical driver event of LIHC [97], were shown to correlate with high *BRPF1* levels [64]. Moreover, mutant p53 variants such us p53^R249S^ and p53^Y220C^ were found to bind BRPF1 regulatory elements and those of other chromatin remodeling genes in LIHC cells [74, 75], pointing out that gain-of-function p53 mutations can drive transcriptional programs that directly regulate histone acetylation.

Additionally, exposure of LIHC HepG2 cell line to endosulfan, a lipophilic insecticide with genotoxic proprieties, induced BRPF1 expression [73], whereas treatment with the cytokine NFα led to its downregulation [78]. These findings indicate that BRPF1 levels can be modulated by environmental and inflammatory stimuli and may play a role in cellular stress and immune-related pathways.

Beyond its overexpression, BRPF1 has been identified as tissue biomarker in LIHC, with elevated levels correlating with poor overall and disease-free survival. Positive correlations have also been reported between BRPF1, MOZ, MORF, and Ki67 [64], as well as with two additional prognostic factors - MTA2 and Gα-interacting vesicle-associated protein [98, 99], suggesting a possible synergistic role in LIHC progression. The association between BRPF1 and Ki67 suggests a role in promoting cell proliferation. Indeed, both genetic knockout and pharmacological inhibition of BRPF1 significantly suppressed tumor growth in vivo [64]. In vitro, treatment with GSK5959 and pan-BRPF inhibitors OF-1 and NI-57 reduced colony formation and proliferation, inducing G1 cell cycle arrest and senescence (Fig. 4A). In vivo, GSK5959 notably suppressed subcutaneous tumor growth. Additionally, BRPF1 was found to be upregulated in CD133+ liver cancer stem cells, whereas its inhibition led to downregulation of *NOTCH1*, *OCT4,* and *EPCAM* in Huh-7 cells, supporting its role in maintaining cancer stemness. Mechanistically, BRPF1 was shown to regulate E2F2 and EZH2 oncogene expression *via* modulation of MOZ/MORF acetyltransferase activity, affecting H3K14 acetylation at their promoters [64].

Concerning other types of gastrointestinal cancers, in COAD, Ling et al. described the Wnt/β-catenin coactivator Pygo2 as an oncogenic driver of cell proliferation and stemness. They uncovered a Pygo2-H3K4me2/3-dependent mechanism that upregulates *BRPF1* by binding its promoter [69] (Fig. 4B). Pygo2 and BRPF1 expression levels were positively correlated, and *BRPF1* knockdown significantly reduced colony formation capacity in Pygo2^high^ cells, while overexpression enhanced growth in Pygo2^low^ cells (Fig. 4B). In vitro and in vivo experiments confirmed that GSK5959-mediated BRPF1 inhibition exerted antiproliferative and anticlonogenic effects in Pygo2^high^ COAD cells [69].

In gastrointestinal stromal tumor (GIST), genome-wide CRISPR-Cas9 screens identified KAT6A, MEAF6, and BRPF1, all components of MOZ HAT complex, as genes with high dependency scores. These genes regulate transcription factors essential for oncogenic signaling and cell-cycle progression. However, BRPF1 inhibition by PFI-4 or GSK6853 did not impair GIST cell proliferation, possibly due to the low drug concentrations used in the study [84], a result inconsistent with findings in other tumor types (summarized in Table 1).

**Table 1:** Summary of the antitumoral effects induced by BRPF1 inhibitors in vitro and in vivo.

Inhibitor	Reference	Tumor type	Cell lines	Treatment time	Drug concentration	Main results
GSK5959	[64]	Hepatocellular carcinoma	MHCC97L, Huh-7, Hep3B, subcutaneous tumor xenograft models	in vitro - 5–6 days; in vivo - 2 weeks	in vitro - 1–10 μM; in vivo - 30 mg/kg/day	Significant reduction of cell proliferation and colony formation in multiple HCC cell lines; significant inhibition of subcutaneous tumor growth without causing any observable toxicity
	[69]	Colon adenocarcinoma	SW480, HT29, COLO-205, SW620, HCT116, HCT15; SW480 and SW620-derived subcutaneous tumor xenograft models	in vitro - 5 days; in vivo – not indicated	in vitro - 10 μM, 20 μM; in vivo – not indicated	Significant reduction of cell proliferation and colony formation in Pygo2^high^ cell lines; Pygo2^high^ SW480-derived tumor xenografts were sensitive to BRPF1 inhibition, whereas Pygo2^low^ SW620-derived tumor xenografts showed mild responses and were more resistant to BRPF1 inhibition.
	[61]	Taxane-resistant prostate cancer	Du145-DtxR, 22Rv1-DtxR	3 days	1.25–5 μM	In co-treatment with docetaxel (Dtx), significant reduction of cell proliferation and colony formation for both resistant cell clones, cell cycle alteration and increased apoptosis
	[68]	Anti-estrogen-sensitive and -resistant luminal-like ERα+ breast cancer	MCF7, MCF7 TAM-R	1–12 days	2.5–20 μM	Significant reduction of cell viability, cell cycle deregulation and altered gene expression profiles
	[76]	Primary and metastatic high-grade serous ovarian cancer	PEO1, PEO4, PEO14, OVCAR-3, Caov-3	3–14 days	10 μM, 20 μM	Significant reduction of cell proliferation, migration and invasion; enhanced apoptosis and DNA damage, cell cycle deregulation and alterations in gene expression profiles
GSK6853	[65]	High-grade glioma	U87-MG, U251	21 days	20 μM, 40 μM, 80 μM	Definition of GSK6853 IC_50_ curve for both cell lines; significant reduction of cell proliferation and colony formation
	[84]	Gastrointestinal stromal tumor	GIST-T1, GIST48B	21 days	0.1 μM, 1 μM	No significant differences were observed in cell growth and proliferation compared to control groups (DMSO only)
	[96]	Hepatocellular carcinoma	Huh7	3 days	1.56–50 μM	Significant reduction of cell viability after treatment with GSK6853 alone and a JQ1/GSK6853 combinatorial treatment using a concentration matrix
	[68]	Anti-estrogen-sensitive and -resistant luminal-like ERα+ breast cancer	MCF7, MCF7 TAM-R, ERα+ patient derived organoids	In vitro -1–12 days, ex vivo – 10 days	In vitro - 2.5–20 μM, ex vivo – 10, 20, 30 μM	Significant reduction of cell viability, altered distribution of cell cycle phases and gene expression profiles
	[76]	Metastatic high-grade serous ovarian cancer	PEO1, PEO4, PEO14, OVCAR-3, Caov-3	3–14 days	10 μM, 20 μM	Significant reduction of cell proliferation, migration and invasion; enhanced apoptosis and DNA damage, cell cycle deregulation and alteration of gene expression profiles
NI-57	[64]	Hepatocellular carcinoma	MHCC97L, Hep3B	5–6 days	25–100 μM	Significant reduction of cell proliferation and colony formation in multiple HCC cell lines
OF-1	[64]	Hepatocellular carcinoma	MHCC97L, Hep3B	5–6 days	10 μM, 20 μM	Significant reduction of cell proliferation and colony formation in multiple HCC cell lines
	[67]	Melanoma	M14, SKMEL-28	5 days	0.1–10 μM	Significant reduction of cell proliferation in both cell lines
	[83]	Melanoma	A375, M14	14 days	0.1–10 μM	Significant reduction of cell proliferation alone or in combination with XL413 (1 μM); enhancement of XL413-mediated suppression of clonogenic activity and anchorage-independent growth
	[61]	Taxane-resistant prostate cancer	Du145-DtxR, 22Rv1-DtxR	3 days	1.25–5 μM	In co-treatment with Dtx, significant reduction of cell proliferation and colony formation for both resistant cell clones, cell cycle alteration and increased apoptosis
PFI-4	[84]	Gastrointestinal stromal tumor	GIST-T1, GIST48B	21 days	0.1 μM, 1 μM	No significant differences were observed in cell growth and proliferation compared to control groups (DMSO only)
	[61]	Taxane-resistant prostate cancer	Du145-DtxR, 22Rv1-DtxR	3 days	1.25–5 μM	In co-treatment with Dtx, significant reduction of cell proliferation and colony formation for both resistant cell clones, cell cycle alteration and increased apoptosis

Lastly, BRPF1 upregulation was also reported in ESCA. In cfDNA samples from ESCA patients, the *BRPF1* promoter, altogether with those of other 48 genes, showed low reads density, suggesting a nucleosome-unprotected chromatin state, a potential marker of transcriptional activity and a candidate for diagnostic and prognostic applications [100].

### The role of BRPF1 in genitourinary tumors

BRPF1 has been implicated in prostate and ovarian cancer development and proposed as a cancer progression biomarker. In PCa, Cevatemre et al. reported low BRPF1 expression, however, its increase has been associated with higher Gleason score, advanced tumor stage, recurrence risk and shorter disease-free survival (DFS) [61, 72]. High BRPF1 expression was also observed in taxane-treated PCa patients, suggesting its utility as a progression marker [61]. *BRPF1* expression, along with *FTH1*, *OSBP*, *PHC3*, and *UACA*, differed in high-, intermediate- and low-risk patients, suggesting their diagnostic potential for early-stage PCa stratification [101].

The diagnostic potential of *BRPF1* was further supported by its expression in urine samples from PCa patients, where BRPF1 mRNA was consistently downregulated in both high- and low-stage PCa versus benign prostate hypertrophy, a common non-malignant condition often challenging to distinguish from PCa at differential diagnosis. Moreover, BRPF1 levels significantly differed between Gleason Score 7 and 8 patients’ groups [101].

Functional studies confirmed the role of BRPF1 in PCa propagation, showing that BRPF1 knockdown impaired PCa cell growth, while overexpression enhanced colony formation [72]. Mechanistically, BRPF1 accumulation in PCa cells enhances its recruitment to *SREBP2* promoter, leading to increased H3K4me3 level and upregulation of *SREBP2* transcription. Conversely, BRPF1 depletion reduces H3K4me3 and SREBP2 expression, indicating an interplay between BRPF1 and SREBP2-driven MVA metabolism in PCa. Moreover, BRPF1 accumulation in PCa may result from stabilization through USP35-mediated deubiquitination, as USP35 is upregulated in PCa and associated with poor prognosis. A positive association between USP35, BRPF1 and SREBP2 was observed in PCa samples, whereas both in vitro and in vivo studies demonstrated that targeting BRPF1/SREBP2/MVA axis endows a therapeutical vulnerability for USP35^high^ PCa [72] (Fig. 4C).

Beyond its role in PCa progression, BRPF1 was implicated in taxane resistance, as shown by Cevatemre et al. who identified BRPF proteins among the top 5 epigenetic targets for resensitizing PCa cell to chemotherapy during a screen comparing taxane-sensitive and -resistant cells [61]. Further analysis demonstrated that BRPF1 pharmacological inhibition reverted taxane resistance by affecting ABCB1-mediated drug efflux and inducing G2/M cell cycle arrest in resistant cells (Fig. 4C). Moreover, BRPF1 pharmacological inhibition synergized with taxanes, reducing viability and colony formation in taxane-resistant PCa cells, potentially via downregulation of ABCB1, whose transcription is directly regulated by BRPF1. Moreover, BRPF1 knockdown also altered mTORC1 and UPR signaling in resistant PCa cells [61].

In ovarian cancer (OC), *BRPF1*genomic alterations, primarily amplifications, were found in 3% of ovarian serous cystadenocarcinoma (OSC) [80]. Although *BRPF1* mRNA levels were downregulated in tumors compared to normal ovaries, protein levels remained unchanged [80]. Conversely, CSIOVDB database analysis showed slightly higher *BRPF1* expression in tumor cells compared to ovarian surface epithelium [76]. These discrepancies may arise from differences in normal sample selection. Notably, increased cytoplasmic BRPF1 expression was observed in OSC, suggesting aberrant nuclear traslocation [80]. This may result from BRPF1 fucosylation, a PTM implicated in OC. Alberto-Aguilar et al. found that culturing OC cells with patients’ ascites enhanced BRPF1 fucosylation, potentially increasing the protein size and hindering the nuclear transfer. However, as other studies suggest the opposite effect, further research is needed [102].

Investigations of BRPF1 prognostic potential in OC patients revealed that high BRPF1 expression correlated with a worse overall survival, an advanced OC stage, and grade [76]. *BRPF1* was also part of the 7-gene prognostic signature established by LASSO regression analysis for OSC, whereas both Cox and Multi-Cox analysis highlighted it as a key risk factor. Finally, BRPF1 mRNA levels accurately predict overall and DFS at 1, 3, and 5 years [80], supporting its value as a prognostic biomarker for OSC patients.

Interestingly, BRPF1 expression negatively correlate with the expression of immune checkpoints *(CD274*, *CTLA4*, *HAVCR2*, *PDCD1LG2*, *SIGLEC15)* and immune cell infiltration by neutrophils and macrophages, while positively correlating with naïve B cells and CD8 + T cells, suggesting a role in immune modulation in OC [80].

Functionally, *BRPF1* knockdown impaired OC cell viability and proliferation by increasing ROS levels and reducing glucose uptake, ATP, and lactate production. It also suppressed Wnt pathway by inhibition of nuclear β-catenin (Fig. 4F). These findings indicate that BRPF1 promotes proliferation, anaerobic metabolism, and Wnt signaling in OC [80]. Moreover, genome-wide CRISPR-Cas9 screening identified *BRPF1, MEN1*, and *KMT2A* as essential for metastatic OC cell survival [76, 103]. Pharmacological inhibition of BRPF1 attenuated HGSOC cell proliferation and invasiveness, induced cell cycle deregulation, apoptosis, and DNA damage (Fig. 4F), and altered metabolic and pro-inflammatory pathways. siRNA- and GSK6853-mediated BRPF1 inhibition commonly deregulated NAD signaling and PPARα-mediated lipid metabolism, which may drive BRPF1 inhibitor efficacy in metastatic HGSOC [76]. Finally, the nonpeptide bradykinin antagonist BKM-570 downregulated *BRPF1* expression, suggesting that its antiproliferative and cytotoxic effect may be partly BRPF1-dependent [79].

*BRPF1* also ranks among the top 25 amplified chromatin regulators in urothelial carcinoma [104]. In endometrial cancer cells, PTEN restoration increased *BRPF1* expression, implicating epigenetic deregulation in the proliferation and differentiation of PTEN-deficient tumors [71].

### The role of BRPF1 in brain cancers

Current literature suggests that BRPF1 acts as an oncogenic factor in certain brain malignancies. Its expression is markedly elevated in high-grade gliomas (HGGs), compared to low-grade gliomas and normal brain tissues. In vitro studies using U87-MG and U251 glioma cell lines revealed that both GSK6853-mediated pharmacologic inhibition and shRNA-mediated silencing of *BRPF1* significant impaired cell proliferation, supporting its potential as an epigenetic therapeutic target in HGGs [65].

In glioblastoma, BRPF1 was identified within a transcriptional regulator gene cluster, also including other epigenetic readers such as *BRD7*, *BRD9*, *SMARCA2*, *SMARCA4* and *TAF1*, that correlated with reduced contrast enhancement on MRI, a marker of less aggressive imaging features [105].

BRPF1 is also involved in adult Sonic hedgehog medulloblastoma (SHH-MB), which frequently harbors mutations in *IDH1* and epigenetic modifiers such as *BRPF1* and *KANSL1* [106]. Recurrent *BRPF1* mutations are observed in adult tumors, while they are absent or very rare in pediatric samples. Additionally, an association between *BRPF1/2/3* and *Smoothened* (SMO) mutations has been described [107], although subsequent evidence identified BRPF1-mutant SHH-MBs without SMO involvement, indicating that BRPF1 may contribute to tumorigenesis through SMO-independent mechanisms [108]. Mechanistic insight was provided by Aiello et al. who demonstrated that co-expression of a truncated BRPF1 and SmoM2, a mutant Smo protein, in postmitotic adult granule neurons induced tumorigenesis via neuronal dedifferentiation and disruption of chromatin accessibility at stemness-associated loci [85].

### The role of BRPF1 in skin tumors

Significant *BRPF1* mRNA overexpression has been reported in melanoma tumors compared to normal skin, based on the Talantov dataset [66]. This result was confirmed by immunohistochemistry data from the Human Protein Atlas, which showed BRPF1 protein expression in melanoma tissues predominantly at 75–25% and <25% levels, with weak staining intensity [67]. Interestingly, comparison of high- and low-risk uveal melanoma samples revealed higher BRPF1 expression in tumors characterized by low-risk disease phenotype [109]. Moreover, elevated expression of *BRPF1*, along with other BrD genes including *TRIM28*, *SMARCA4*, *BRD4*, has been associated with shorter overall survival in patients with metastatic melanoma [77]. A pathogenic frameshift somatic mutation in *BRPF1* (p.Q938fs), was also identified in 1 out of 17 *BRAF* mutated, Spitzoid-type melanocytic tumors [110], suggesting a possible role in melanoma pathogenesis.

Pharmacological inhibition of BRPF1 by OF1 significantly decreased cell survival rate in BRAF-mutant melanoma cells [77]. Furthermore, combinatorial inhibition of BRPF1 and CDC7, a kinase overexpressed in melanoma and linked to poor prognosis, enhanced the anticancer effect of the CDC7 inhibitor XL413 in vitro and in vivo. Mechanistically, the combined treatment suppressed MYC and IGF1R oncogenic pathways, enhanced apoptosis, and induced cell cycle arrest at G2/M phase [83] (Fig. 4D).

### The role of BRPF1 in leukemia

Acute myeloid leukemia is a hematopoietic disorder characterized by a broad spectrum of chromosomal abnormalities and gene mutations. Chromosomal translocations in AML often generate fusion genes that promote aberrant transcriptional activation [111, 112]. Among these, translocations involving *MOZ* gene and resulting in the *MOZ-TIF2* fusion are often associated with an adverse prognosis. BRPF1 was implicated in the pathogenesis of *MOZ-TIF2-*driven AML. Specifically, BRPF1 acts as a direct regulator of *HOX* genes, such as *Hoxa9*, *Hoxa10,* and Meis1, which are commonly overexpressed in AML and contribute to the immortalization of bone marrow cell progenitors [82]. This was demonstrated by shRNA-mediated *BRPF1* knockdown in AML cells, which led to a significant reduction of *Hoxa9*, *Hoxa10*, and *Meis1* expression, as well as impaired colony-forming ability, effect rescued by BRPF1 re-expression. Moreover, BRPF1 was found to colocalize with MOZ on chromatin regions of *Hoxa9* and *Hoxa10* loci, and its depletion reduced MOZ occupancy at these sites. These findings suggest that BRPF1 directs the recruitment of MOZ to *HOX* gene promoters, thereby enhancing local histone H3 acetylation and transcriptional activation (Fig. 4E). In line with these observations, Yan et al. demonstrated that MOZ regulates AML cell differentiation through epigenetic mechanisms requiring BRPF1, further highlighting their functional co-dependency in AML pathology [113]. More recently, in *NUP98*-rearranged AML, a subtype commonly observed in childhood leukemia, BRPF1 was identified as a chromatin-associated partner of MYST family HAT complex components MOZ and HBO1 and shown to interact with NUP98 fusion oncoproteins. In an in vivo CRISPR/Cas9 screen targeting epigenetic regulators in NUP98:KDM5A-expressing hematopoietic stem and progenitor cells, *BRPF1* emerged among the top hits, alongside genes involved in stem cell division and histone acetylation. This finding was further validated by CRISPR-based competition assay, which underscored the essential roles of MYST family HAT complexes in sustaining leukemogenesis driven by *NUP98* rearrangements [114].

### The role of BRPF1 in breast cancer

BRPF1 has been implicated in other tumor types, notably breast. Salvati et al. identified BRPF1 as a critical epigenetic regulator in luminal-like Estrogen Receptor α (ERα)⁺BC. Through integrative analyses combining genome-wide dropout screenings and siRNA-mediated gene knockdown, they demonstrated that BRPF1 is essential for the proliferation and survival of both AE-sensitive and AE-resistant breast cancer cells. Mechanistically, BRPF1 was shown to interact with ERα on chromatin, promoting transcriptional programs linked to cell cycle progression. Moreover, BRPF1 directly regulates ERα expression, further sustaining estrogen signaling in ERα⁺ breast cancer cells. Its pharmacological inhibition with GSK5959 or GSK6853 led to decreased proliferation, increased apoptosis, and altered chromatin accessibility, both in AE-sensitive and AE-resistant BC cell lines and patient-derived organoids, suggesting BRPF1 as a promising therapeutic target to overcome endocrine therapy resistance in BC [68] (Fig. 4G). Moreover, BRPF1 has emerged as a predictive biomarker. Its co-expression with KIAA0831 contributed to the definition and validation of CTSP-6, a multigene signature predictive of pathological complete response in BC patients undergoing pre-operative taxane/anthracycline-based chemotherapy [115].

The therapeutic relevance of BRPF1 has also been demonstrated in triple negative BC (TNBC), where it was identified as a regulator of Taxol resistance. In resistant TNBC models, BRPF1 knockout or inhibition with PFI-4 or OF-1 significantly reduced cell viability. Mechanistic studies revealed a dual contribution to drug resistance: upregulation of ribosome biogenesis genes and direct activation of ABCB1, encoding an ATP-dependent drug efflux pump. [86].

### The role of BRPF1 in other cancers

In respiratory tumors, a frequent deletion in 3p25.3 cytoband, encompassing the *BRPF1* locus, was reported in a subset of non-small cell lung cancers [116]. In lung adenocarcinoma, BRPF1 was found to be a target of hsa-mir-122, a microRNA often up-regulated in this cancer type and associated with poor prognosis [117].

Finally, genome-wide CRISPR-Cas9 knockout screening showed that *BRPF1*, along with *HBO1*, *BRPF2* and *MEAF6*, was essential for the growth and survival of EBV-positive nasopharyngeal carcinoma cells but dispensable in normal nasopharyngeal epithelial cells, suggesting their potential essential role in NPC development [81].

Overall, the oncogenic potential of BRPF1 emerges across a wide spectrum of malignancies, where it contributes to tumor progression through diverse mechanisms including transcriptional regulation of oncogenes, chromatin remodeling, stemness maintenance, and metabolic reprogramming. From liver and colorectal cancers to prostate, ovarian, breast, and hematologic malignancies, BRPF1 is frequently upregulated, correlates with poor prognosis, and sustains key tumor hallmarks such as proliferation, resistance to therapy, and immune evasion. Its involvement in lineage-specific pathways—as exemplified by the BRPF1/SP1/p53 axis in LIHC, the BRPF1/SREBP2/MVA circuit in PCa, or the BRPF1/ERα signaling in BC—highlights its multifaceted role in cancer biology. These findings support the notion that BRPF1 is not only a biomarker of aggressive disease but also a driver of oncogenic programs that may be exploited therapeutically.

## Conclusions and future perspectives

The dynamic modulation of the cancer epigenome has emerged as a central theme in molecular oncology, leading to the development of numerous small-molecules epigenetic drugs (“epidrugs”), as promising alternatives or complements to conventional chemotherapies [118]. BRPF1, a chromatin reader and scaffold protein within several HAT complexes, has generated increasing attention for its critical roles in transcriptional regulation through recognition of specific HPTMs.

A comprehensive review of current literature, complemented by data from tissue expression databases, highlights BRPF1 as a key regulator in multiple cancer types. Its oncogenic activity has been documented in breast, prostate, ovarian, and liver cancers, as well as in gliomas, leukemia, and nasopharyngeal carcinoma. Mechanistically, BRPF1 contributes to tumorigenesis by regulating genes involved in hormone signaling (ERα), lipid metabolism (SREBP2), chromatin remodeling (EZH2, HOX genes), and drug resistance (ABCB1), supporting proliferation, stemness, and survival under therapeutic pressure (see Fig. 4).

Importantly, pharmacological inhibition of BRPF1 using selective bromodomain inhibitors (e.g., GSK5959, GSK6853, OF-1, PFI-4) has shown promising preclinical antitumor efficacy, both as monotherapy and in combination with chemotherapeutics or endocrine agents, across various tumor models, including AE-resistant breast cancer, Taxol-resistant triple-negative breast cancer, hepatocellular carcinoma, ovarian cancer and AML.

Despite these advances, the translation of BRPF1-targeted strategies into the clinical practice is still at an early stage. To date, no BRPF1-specific inhibitors have reached clinical trials, and their safety, selectivity, and pharmacokinetic profiles remain insufficiently characterized. Further investigations are needed to validate their efficacy in clinically related models, such as patient-derived xenografts and organoids, and to identify the tumor contexts most likely to benefit from BRPF1 blockade.

In parallel, a deeper understanding of BRPF1 protein partners, context-specific functions, and transcriptional targets will be essential for developing effective combinatorial strategies and precision-based therapeutic approaches. Taken together, current evidence supports BRPF1 as a compelling and versatile target in cancer epigenetics, warranting further preclinical and translational efforts to harness its therapeutic potential.
